# Risk of Sudden Infant Death Syndrome Among Siblings of Children Who Died of Sudden Infant Death Syndrome in Denmark

**DOI:** 10.1001/jamanetworkopen.2022.52724

**Published:** 2023-01-25

**Authors:** Charlotte Glinge, Sára Rossetti, Louise Bruun Oestergaard, Niels Kjær Stampe, Thomas Hadberg Lynge, Regitze Skals, Bo Gregers Winkel, Elisabeth M. Lodder, Connie R. Bezzina, Gunnar Gislason, Jytte Banner, Elijah R. Behr, Christian Torp-Pedersen, Reza Jabbari, Jacob Tfelt-Hansen

**Affiliations:** 1 Department of Cardiology, The Heart Centre, Copenhagen University Hospital, Rigshospitalet, Copenhagen, Denmark; 2 Department of Clinical and Experimental Cardiology, Heart Centre, Amsterdam Cardiovascular Sciences, Amsterdam UMC, University of Amsterdam, Amsterdam, the Netherlands; 3 Department of Cardiology, Copenhagen University Hospital–Herlev and Gentofte, Copenhagen, Denmark; 4 Department of Cardiology, Aalborg University Hospital, Aalborg, Denmark; 5 The Danish Heart Foundation, Copenhagen, Denmark; 6 Department of Forensic Medicine, Copenhagen University Hospital, Rigshospitalet, Copenhagen, Denmark; 7 Cardiology Clinical Academic Group, Cardiology Section, St George’s, University of London, London, United Kingdom; 8 St George’s University Hospitals NHS Foundation Trust, London, United Kingdom; 9 Mayo Clinic Healthcare, London, United Kingdom; 10 Department of Cardiology, North Zealand University Hospital, Hillerød, Denmark; 11 Department of Public Health, University of Copenhagen, Denmark

## Abstract

**Importance:**

Sudden infant death syndrome (SIDS) remains a leading cause of death during the first year of life. The etiology of SIDS is complex and remains largely unknown.

**Objective:**

To evaluate whether siblings of children who died of SIDS have a higher risk of SIDS compared with the general pediatric population.

**Design, Setting, and Participants:**

This register-based cohort study used Danish nationwide registers. Participants were all infants (<1 year) in Denmark between January 1, 1978, and December 31, 2016, including siblings of children who died of SIDS. Siblings were followed up from the index cases’ date of SIDS, date of birth, or immigration, whichever came first, and until age 1 year, emigration, developing SIDS, death, or study end. The median (IQR) follow-up was 1 (1-1) year. Data analysis was conducted from January 2017 to October 2022.

**Main Outcomes and Measures:**

Standardized incidence ratios (SIRs) of SIDS were calculated with Poisson regression models relative to the general population.

**Results:**

In a population of 2 666 834 consecutive births (1 395 199 [52%] male), 1540 infants died of SIDS (median [IQR] age at SIDS, 3 [2-4] months) during a 39-year study period. A total of 2384 younger siblings (cases) to index cases (first sibling with SIDS) were identified. A higher rate of SIDS was observed among siblings compared with the general population, with SIRs of 4.27 (95% CI, 2.13-8.53) after adjustment for sex, age, and calendar year and of 3.50 (95% CI, 1.75-7.01) after further adjustment for mother’s age (<29 years vs ≥29 years) and education (high school vs after high school).

**Conclusions and Relevance:**

In this nationwide study, having a sibling who died of SIDS was associated with a 4-fold higher risk of SIDS compared with the general population. Shared genetic and/or environmental factors may contribute to the observed clustering of SIDS. The family history of SIDS should be considered when assessing SIDS risk in clinical settings. A multidisciplinary genetic evaluation of families with SIDS could provide additional evidence.

## Key Points

**Question:**

Is a family history of sudden infant death syndrome (SIDS) associated with increased risk of subsequent SIDS compared with the general population?

**Findings:**

In this cohort study of more than 2.6 million consecutive births in Denmark between 1978 and 2016, a total of 1540 infants died of SIDS. A higher rate of SIDS was observed among siblings of children who died of SIDS compared with the general population.

**Meaning:**

These findings suggest that any sibling of a child who died of SIDS should be investigated with great care to exclude genetic and environmental factors.

## Teaser

This cohort study evaluates whether having a sibling who died of sudden infant death syndrome (SIDS) is associated with increased risk of SIDS compared with the general pediatric population among infants in Denmark.

## Introduction

Although sudden infant death syndrome (SIDS) is a leading cause of natural death during the first year of life,^1,2,3^ the underlying mechanisms causing sudden death remain largely unknown, and prevention is still a challenge. SIDS is a diagnosis of exclusion applied to the death of an infant younger than 1 year after an extensive postmortem investigation.^4^ Causes of SIDS are considered to be complex and multifactorial.^5^ There is a triple-risk hypothesis with 3 risk factors that predispose to SIDS: (1) an exogenous stressor (eg, sleeping prone, smoke exposure), (2) a critical development period (usually age 2-4 months), and (3) an underlying vulnerability, such as genetic susceptibility.^6,7^ To date, it is not possible to predict infants at risk of SIDS. Environmental factors may be risk factors in an infant who is genetically susceptible and quite innocuous to a nonsusceptible infant. A positive familial aggregation study is an important step to suggest the usefulness of clinical genetic work-up of the families. Even though positive family history of SIDS has been described,^8,9,10,11,12,13,14^ it is unknown whether a familial aggregation exists in the general population. Previous studies of familial aggregation were performed on small and/or selected populations^8,9,10,11,12^; therefore, a nationwide study on an unselected pediatric population with information on family history is warranted to establish a familial clustering of children who died of SIDS. More knowledge is warranted because an association between a family history of SIDS and subsequent SIDS will have public health implications to identify children at risk of SIDS, with the goal of preventing of further SIDS within families. Thus, the aim of this study was to evaluate whether siblings of children who died of SIDS have a higher risk of SIDS compared with the general pediatric population. We hypothesized that the risk of SIDS would be increased among siblings of children who died of SIDS.

## Methods

The study was approved by the Danish Data Protection Agency. In Denmark, retrospective register-based studies in which individuals cannot be identified do not require approval from the ethics committees. Conduct of this study was in accordance with the Strengthening the Reporting of Observational Studies in Epidemiology (STROBE) recommendations.^15^ Informed consent is not required for administrative registry studies in Denmark. All data were encrypted, and Statistics Denmark hosted and linked the data.

### Data Sources

We conducted this nationwide study based on health and administrative registries in Denmark. In Denmark, all residents are given a permanent and unique civil registration number at birth or immigration that enables individual-level linkage between all health and administrative registries.^16^ Since 1954, there is close to complete registration of parents and siblings to individuals born in Denmark as well as complete data on hospital diagnosis and cause of death. Using the Danish Cause of Death Register, we identified all persons who had died of SIDS.^17^ SIDS is a diagnosis of exclusion and defined as the sudden death of an infant younger than 1 year, which remains unexplained after a thorough autopsy, including investigation of the scene of death and review of the medical history.^4,5,18^ In Denmark, it is mandatory by law to complete death certificates. Danish death certificates are well suited as a primary screening tool for identifying sudden and unexpected deaths due to a detailed description of the circumstances of the death, including a summary of the death scene investigation on the certificate. Autopsies have to be performed if the external examination cannot establish the mode of death or at the request of physicians or relatives. Information from the autopsy reports, including toxicology examinations, is also added to the death certificate. We used the *International Classification of Diseases, Eighth Revision* (*ICD-8*) for deaths before 1999 (*ICD-8* code 795) and *International Statistical Classification of Diseases and Related Health Problems, Tenth Revision *(*ICD-10*) for deaths since 1999 (*ICD-10* code R95). Our group has previously validated the Cause of Death Registry by comparing the cause of death after reading the autopsy report and the official *ICD-10* diagnosis denoting SIDS (R95) in the registry between 2000 and 2006.^3,19^ The Cause of Death Registry correctly categorized 81 of 98 SIDS cases (83%). Furthermore, we used the Danish Medical Birth Registry to identify all siblings of children who died of SIDS.^20^ The first sibling in a sibship (group of siblings with the same mother) who died of SIDS was named the index case. Information on vital status (date of birth, date of death, sex, emigration, and immigration) was obtained from the Danish Civil Registration System,^21^ and data on education and household income were obtained from the Population’s Education Register and the Income Statistics Register, respectively.^22,23^

### Study Population

Using data from the national Civil Registration System, we initially identified all the births in Denmark between January 1, 1978, and December 31, 2016. By cross-linking the Civil Registration System with the Cause of Death Register, all infants who died of SIDS (*ICD-8* code 795; *ICD-10* code R95) and were younger than 1 year at the time of death were included. Using the Medical Birth Registry, we identified all siblings of these children who died of SIDS. Siblings were followed up from the index cases’ date of SIDS, date of birth, or immigration, whichever came first, and until 1 year of age, emigration, developing SIDS, death, or study end (December 31, 2016). Persons who died before January 1, 1978, stillborn babies, and adopted children were excluded. In a sensitivity analysis, we limited the study period to January 1, 2002, and December 31, 2016.

### Socioeconomic Status

The mothers’ educational status was obtained from the Population’s Education Register, which contains data on current and highest completed education authorized by the Danish Ministry of Education.^22^ This register is reported by the Statistics Denmark as having a high validity.^22^ The highest level of completed education the same year of birth was classified according to UNESCO’s guidelines for classifying education, the International Standard Classification of Education (ie, elementary school, high school, vocational education, short- or medium-length higher education, and longer higher education or research). In addition, household income was collected from the Danish Income Statistics Registry, which is provided by the Danish Tax authorities.^23^ Median household income to the year of birth was calculated and graded in tertiles.

### Statistical Analysis

Descriptive data were reported as frequencies and percentages or medians with IQRs, as appropriate. Baseline characteristics were summarized, and differences between groups were tested by applying the χ^2^ test for categorical variables. The relative risk of SIDS among siblings was estimated by Poisson regression modeling, adjusted for sex, age, and calendar year, in which the rate of SIDS in the general pediatric population was used as a fixed reference. The index cases did not participate in the analysis. This method has previously been used by our group.^24,25^ Results are shown as standardized incidence ratios (SIRs) with 95% CIs. As in many other countries, the Danish Health and Medicines Authority recommends supine- or side-sleeping position. To account for risk-intervention campaigns in Denmark, we performed a sensitivity analysis and included only SIDS after year 1992.

All data management and statistical analyses were performed using SAS version 9.4 (SAS Institute) and R version 3.4 (R Project for Statistical Computing) (from Statistic Denmark’s research servers). For all analyses, a 2-sided *P* ≤ .05 was considered statistically significant.

## Results

Through nationwide health and administrative registries, we included all infants (2 666 834; 1 395 199 [52%] male) younger than 1 year between 1978 and 2016 in Denmark. We identified 1540 infants who died of SIDS during the 39-year study period, of whom 1465 had family information. We identified 2384 siblings of these 1465 SIDS index cases (Figure). Most cases of SIDS (1324 [90%]) occurred within the first 6 months of life (median [IQR] age at SIDS, 3 [2-4] months), and boys represented 61% of these children (888 infants). Table 1 shows the clinical characteristics of siblings of SIDS index cases compared with the general pediatric population. There were no significant differences regarding sex and mother’s age at childbirth between the 2 groups. However, siblings of SIDS index cases were more likely to live in households with low income (851 [50%] vs 593 580 [33%]) and to have mothers with only elementary school education (1216 [54%] vs 559 802 [26%]) compared with the general population. Clinical characteristics of the index cases are shown in eTable 1 in Supplement 1.

**Figure.**
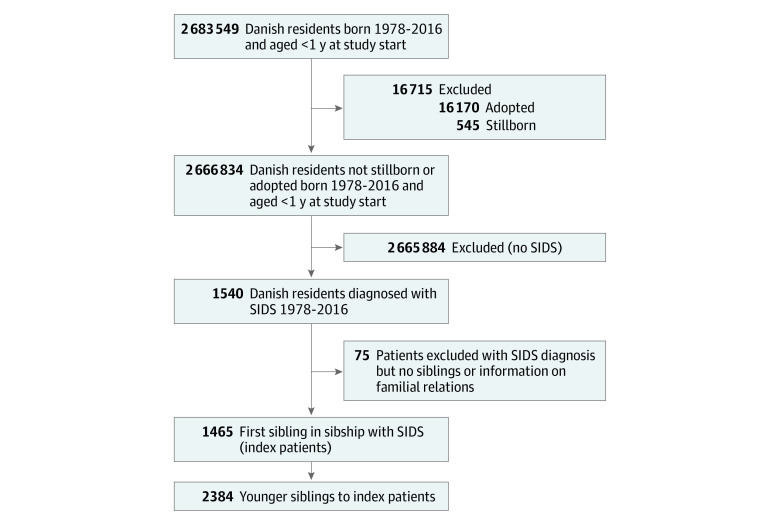
Flowchart Illustrating the Selection of the Sibling Cohort, Which Comprised Siblings to Infants With Sudden Infant Death Syndrome (SIDS) Between 1978 and 2016

**Table 1.** Baseline Characteristics of Siblings of Index Cases Compared With the General Pediatric Population

Characteristic	Infants, No. (%)		*P* value
	Siblings of index cases (n = 2384)	General population (n = 2 666 834)	
Sex			
Male	1223 (51)	1 395 199 (52)	.32
Female	1161 (49)	1 271 185 (48)	
Maternal age, median (IQR), y	29 (26-33)	29 (26-33)	.53
Household income^a^			
Low	851 (50)	593 580 (33)	<.001
Middle	593 (35)	611 575 (34)	
High	255 (15)	593 584 (33)	
Missing data	685	868 095	
Maternal education			
Elementary	1216 (54)	559 802 (26)	<.001
High school	130 (6)	217 456 (10)	
Vocational	533 (24)	654 019 (31)	
Short- or medium-term higher education	304 (14)	534 239 (25)	
Longer-term higher education	62 (3)	161 477 (8)	
Missing data	139	539 223	

^a^ Household income stratified in tertiles low (<467 031 DKK [US $66 641]), middle (467 032-671 436 DKK [US $66 641-$95 807]), and high (>671 436 DKK [US $95 807]).

After 1 year of follow-up, a total of 8 siblings died of SIDS at a median (IQR) age of 2.5 (1.4-4.1) months. The SIR calculated by the Poisson model was 4.27 (95% CI, 2.13-8.53) after adjustment for sex, age, and calendar year and 3.50 (95% CI, 1.75-7.01) after further adjustment for mother’s age (<29 years vs ≥29 years) and education (high school vs after high school) (Table 2). Furthermore, we performed a sensitivity analysis and included only SIDS deaths after 1992. Additional sensitivity analyses including SIDS deaths during 2002 to 2016 (122 younger siblings to 92 index cases) were carried out and remained significant (eFigure and eTables 2 and 3 in Supplement 1). The SIRs calculated by the Poisson model were 3.94 (95% CI, 2.55-5.32) after adjustment for sex, age, and calendar year and 4.17 (95% CI, 2.21-6.13) after further adjustment for mother’s age (<30 years vs ≥30 years) and education (high school vs after high school) (eTable 4 in Supplement 1). Table 3 shows the clinical characteristics of the 8 siblings who died of SIDS.

**Table 2.** SIRs for SIDS in Sibling of SIDS Index Cases

Group	No. (No. of events)	SIR (95% CI)	
		Model 1^a^	Model 2^b^
General population	2 666 834 (1540)	[Reference]	[Reference]
Siblings of children with SIDS	2384 (8)	4.27 (2.13-8.53)	3.50 (1.75-7.01)
Siblings born after 1992	1286 (4)	9.10 (3.42-24.25)	7.27 (2.73-19.38)

Abbreviation: SIDS, sudden infant death syndrome; SIR, standardized incidence ratio.

^a^ Adjusted for age, sex, and calendar year.

^b^ Adjusted for age, sex, calendar year, maternal age (<29 y [median age] vs ≥29 y), and maternal education (high school vs after high school).

**Table 3.** Baseline Characteristics of Siblings to SIDS Index Cases Who Died of SIDS

Characteristic	Siblings to children who died of SIDS, median (IQR) (n = 8)
Death age, mo	2.5 (1.5-4.1)
Sex, No. (%)	
Male	4 (50)
Female	4 (50)
Mothers age, y	28 (25-30)
Time between index case death and sibling death, y	3 (2-4)

Abbreviation: SIDS, sudden infant death syndrome.

## Discussion

Our study includes one of the largest cohorts of unselected children who died from SIDS and their siblings at risk for potential SIDS, and in line with our hypothesis, we found a familial aggregation of occurrence of SIDS: siblings of SIDS victims had a 4-fold higher risk of SIDS compared with the general population. Whether this familial aggregation is a consequence of shared environmental risk factors, genetic factors, or a combination remains unknown, but our findings emphasize the importance of including a detailed family history of SIDS to identify siblings at risk of SIDS, with the goal to prevent further SIDS within families. Any child who died of SIDS who has a sibling should be investigated with great care to exclude genetic and/or environmental factors.

To date, the best established risk factors, or trigger events, are environmental risk factors.^26^ But despite a great reduction in the SIDS rate by targeting these environmental risk factors, SIDS remains a leading cause of natural death during the first year of life, and SIDS rates have plateaued in recent years,^1^ suggesting that other factors play a role. In the early 1990s, the prone sleep position was identified as a major risk factor for SIDS.^27^ Subsequent public health campaigns reduced the incidence of prone sleeping in infants, and follow-up studies showed that this change in behavior was associated with a dramatic reduction in the SIDS rate.^28,29,30,31,32,33^ We performed a sensitivity analysis to evaluate whether prone sleeping alone accounted for familial aggregation. We found familial aggregation of SIDS after 1992, suggesting that environmental risk factors alone are unlikely to be the leading cause of the observed familial aggregation.^34^ In addition, although studies have shown associations between SIDS and smoke exposure^35,36^ and lower socioeconomic status,^29,37^ families of nonsmoking parents in higher socioeconomic categories continue to be affected, suggesting that shared genetic factors between siblings contribute to the observed familial aggregation. The aggregation of SIDS in families is the first clue for an underlying genetic susceptibility. This agrees with several studies that have indicated that genes involved in inherited cardiac diseases and metabolic disorders play a role in SIDS.^38,39,40,41,42^ During the past few years, genetic research has significantly improved our understanding of the genetic basis of SIDS,^41,43,44,45^ but the magnitude of its contribution and the mode of inheritance are still obscure. A sequencing study of 161 infants who died of SIDS identified potentially causative gene variants in 20% of the children, and these were associated with inherited cardiac disorders and metabolic diseases.^41^ Furthermore, data suggests that approximately 5% to 15% of SIDS deaths may stem from pathogenic variations in genes coding for cardiac ion channels associated with long QT syndrome, Brugada syndrome, and catecholaminergic polymorphic ventricular tachycardia.^46,47^ Others have also implicated abnormalities in sodium channels in the skeletal muscle,^44^ nicotine metabolizing enzymes, and regulation of the autonomic nervous system^48^ as promising genetic candidates that may explain SIDS. In addition, there has shown to be a potential overlap with genetic risk of sudden unexpected death in epilepsy and sudden unexplained death at a young age.^49^ Studies have also reported genes involved in the regulation of the immune system as contributors to SIDS risk,^50^ which suggests that the way children who die of SIDS respond to infection—and perhaps not the infection itself—is important.^51^ To date, only 1 published exome study of 427 children who died of SIDS has taken an unbiased approach to investigate a monogenic basis of SIDS.^52^ However, this study did not yield any statistically significant results. Importantly, this does not suggest a lack of genetic contribution to SIDS; rather, it supports an extreme heterogeneity in the genetic causes which cannot be singled out by these methods in a cohort of this size (427 infants). Moreover, an investigation of all previously implicated noncardiac genes underpinning SIDS also failed to show any significant associations of ultrarare or novel variations consistent with autosomal dominant and recessive inheritance patterns.^45^ Therefore, it still needs to be investigated whether infant vulnerability to sudden death may be supported by a more complex polygenic inheritance model. Heritability may not be due to monogenic variants but to low frequency and common genetic variations. Even though we could not distinguish between genetic and shared environmental factors or their interactions in this study, our results and previous studies^8,9,10,11,12^ provide strong support for genetic testing of SIDS index cases to prevent future SIDS in affected families. Although genetic conditions are often inherited, this is not always the case. Several SIDS reports have identified de novo genetic variants.^53,54^ However, our finding of aggregation in the families of children who die of SIDS implies that inheritance plays a role in SIDS, which has important clinical implications for the surviving family because it warrants both clinical screening of family members as well as targeted genetic analysis.

### Recommendations and Future Research

SIDS is a challenge for clinicians with regard to the counselling and future management of possible risk to other family members. To date, prevention is hindered by our inability to identify infants who are at risk of dying suddenly. While there is currently nothing that can guarantee protection from SIDS in an infant, the most effective way described so far to prevent SIDS is through reduction of known environmental risk factors,^55^ so focus should remain on implementation of measures that have already been proven to prevent SIDS. However, the vast majority of infants who sleep in prone position, are exposed to tobacco smoke, and get overheated during sleep do not die in infancy, and numerous infants with none of these risk factors have died of SIDS, suggesting that the etiology is more complex, a view that is supported by our finding of familial aggregation and other studies showing involvement of various genetic pathways.^5,38,40,41,42^ Based on the latter studies, the Heart Rhythm Society/European Heart Rhythm Association/Asia Pacific Heart Rhythm Society recommend molecular autopsy to uncover potential inherited cardiac conditions, and if positive, they recommend that family members are investigated.^56,57^ We agree that a combined approach of molecular autopsy and familial evaluation, with detailed family history, provides the best chance of identifying an inherited condition in the family of multiple children who died of SIDS. Moreover, we believe the best way to find inherited cardiac disease is to have dedicated cardiac genetic clinics with effective family screening programs to ensure thorough cascade family screening. To date, routine infant electrocardiogram (ECG) screening is controversial.^58,59^ Most pediatric cardiologists do not consider such ECG screening to be informative, nor do we. We believe that mass infant ECG screening will have little or no impact on the incidence of SIDS.

In future research, large studies with good statistical power are clearly needed given that SIDS is a rare event, even among relatives of children who died of SIDS. In addition, we believe that important tasks in SIDS research today are to determine the genetic component, the genes that are involved, and the molecular mechanism contributing to SIDS.

### Strengths and Limitations

The strengths of our study lie in the fact that it is so far among the largest studies on SIDS and the follow-up data are complete. All data were retrieved from nationwide registries, guaranteeing reliable estimation of familial risk because the data on family relationships and SIDS were obtained from registered sources with almost complete coverage.

This study has limitations even though confounding factors, including age, gender and socioeconomic status were adjusted in the analysis. The first limitation of our study is the fact that the SIDS diagnosis is an exclusion diagnosis, and even though a great effort has been made to standardize it, it is possible that children who die from SIDS were not investigated sufficiently for a conclusive SIDS diagnosis. Moreover, the definition of SIDS has changed over time, and the autopsy algorithm has advanced.^18^ All other possible causes of death must be excluded for this diagnosis to be made. It is estimated that an alternative diagnosis could have been made in 25% of SIDS cases.^60^ In addition, there should always be a concern for child abuse (ie, intentional suffocation).^61^ Second, we are aware of the fact that the number of SIDS deaths is relatively small, but despite this we believe that the results from this study are relevant, since SIDS cannot solely be explained by environmental factors, and our finding of familial aggregation provides support for additional genetic involvement. Third, our study only addresses family aggregation, and we had limited access to other risk factors. It would have been preferable to control for sleeping environment and smoking exposure, as these are factors that may affect SIDS risk in families. In addition, we know that lower socioeconomic status and lack of education are associated with higher pregnancy-related smoking rates.^62,63^ But we would like to underscore that Denmark is a country with one of the lowest infant mortality rates in Europe,^64^ and a study from Sweden, a comparable neighbor country, has shown that nonsupine sleep position rates are less than 6% and maternal smoking rates are less than 10%.^65^ Nevertheless, although these numbers are relatively low, they could be further reduced by effective communication regarding modifiable risk factors for SIDS and recommendations to reduce unsafe sleeping environment and exposure to cigarette smoke, especially in target populations.

## Conclusions

This study found familial aggregation of SIDS deaths in a large population-based study. Shared genetic factors may contribute to this familial aggregation of SIDS, but the contribution of shared environmental factors cannot be neglected. Although SIDS is very rare and declining, thousands of infants still die of SIDS annually in Europe and the United States,^64^ and answers are still needed to prevent these deaths. Despite ongoing research on the genetic causes of SIDS, there are no definitive answers yet; therefore, we recommend that parents follow the public health guidelines^66^ and that clinicians consider the family history of SIDS when assessing SIDS risk and when implementing preventive interventions.

## Notes

Supplement 1. **eTable 1.** Baseline Characteristics of SIDS Index Cases Compared With the General Pediatric Population During Study Period, 1978-2016 **eTable 2.** Baseline Characteristics of Siblings of Index Cases Compared With the General Pediatric Population During Study Period, 2002-2016 **eTable 3.** Baseline Characteristics of SIDS Index Cases Compared With the General Pediatric Population During Study Period 2002-2016 **eTable 4.** SIRs for SIDS in Siblings of SIDS Index Cases During Study Period 2002-2016 **eFigure.** Flowchart Illustrating the Selection of the Sibling Cohort, Which Comprised Siblings to Index Cases With SIDS Between 2002 and 2016

Supplement 2.
Data Sharing Statement

## References

[1] Matthews TJ, MacDorman MF, Thoma ME. Infant mortality statistics from the 2013 period linked birth/infant death data set. Natl Vital Stat Rep. 2015;64(9):1-30. 26270610

[2] Hakeem GF, Oddy L, Holcroft CA, Abenhaim HA. Incidence and determinants of sudden infant death syndrome: a population-based study on 37 million births. World J Pediatr. 2015;11(1):41-47. doi:10.1007/s12519-014-0530-9 25447630

[3] Winkel BG, Holst AG, Theilade J, . Sudden unexpected death in infancy in Denmark. Scand Cardiovasc J. 2011;45(1):14-20. doi:10.3109/14017431.2010.538433 21133644

[4] Krous HF, Beckwith JB, Byard RW, . Sudden infant death syndrome and unclassified sudden infant deaths: a definitional and diagnostic approach. Pediatrics. 2004;114(1):234-238. doi:10.1542/peds.114.1.234 15231934

[5] Baruteau AE, Tester DJ, Kapplinger JD, Ackerman MJ, Behr ER. Sudden infant death syndrome and inherited cardiac conditions. Nat Rev Cardiol. 2017;14(12):715-726. doi:10.1038/nrcardio.2017.129 28880023

[6] Filiano JJ, Kinney HC. A perspective on neuropathologic findings in victims of the sudden infant death syndrome: the triple-risk model. Biol Neonate. 1994;65(3-4):194-197. doi:10.1159/000244052 8038282

[7] Spinelli J, Collins-Praino L, Van Den Heuvel C, Byard RW. Evolution and significance of the triple risk model in sudden infant death syndrome. J Paediatr Child Health. 2017;53(2):112-115. doi:10.1111/jpc.13429 28028890

[8] Guntheroth WG, Spiers PS. The triple risk hypotheses in sudden infant death syndrome. Pediatrics. 2002;110(5):e64. doi:10.1542/peds.110.5.e64 12415070

[9] Beal SM, Blundell HK. Recurrence incidence of sudden infant death syndrome. Arch Dis Child. 1988;63(8):924-930. doi:10.1136/adc.63.8.924 3415329 PMC1778968

[10] Oyen N, Skjaerven R, Irgens LM. Population-based recurrence risk of sudden infant death syndrome compared with other infant and fetal deaths. Am J Epidemiol. 1996;144(3):300-305. doi:10.1093/oxfordjournals.aje.a008925 8686699

[11] Carpenter RG, Waite A, Coombs RC, . Repeat sudden unexpected and unexplained infant deaths: natural or unnatural? Lancet. 2005;365(9453):29-35. doi:10.1016/S0140-6736(04)17662-9 15639677

[12] Oren J, Kelly DH, Shannon DC. Familial occurrence of sudden infant death syndrome and apnea of infancy. Pediatrics. 1987;80(3):355-358. doi:10.1542/peds.80.3.355 3627886

[13] Beal S. Sudden infant death syndrome in twins. Pediatrics. 1989;84(6):1038-1044. doi:10.1542/peds.84.6.1038 2587132

[14] Garstang JJ, Campbell MJ, Cohen MC, . Recurrent sudden unexpected death in infancy: a case series of sibling deaths. Arch Dis Child. 2020;105(10):945-950. doi:10.1136/archdischild-2019-318379 32527717

[15] von Elm E, Altman DG, Egger M, Pocock SJ, Gøtzsche PC, Vandenbroucke JP; STROBE Initiative. The Strengthening the Reporting of Observational Studies in Epidemiology (STROBE) statement: guidelines for reporting observational studies. Epidemiology. 2007;18(6):800-804. doi:10.1097/EDE.0b013e3181577654 18049194

[16] Thygesen LC, Daasnes C, Thaulow I, Brønnum-Hansen H. Introduction to Danish (nationwide) registers on health and social issues: structure, access, legislation, and archiving. Scand J Public Health. 2011;39(7)(suppl):12-16. doi:10.1177/1403494811399956 21898916

[17] Helweg-Larsen K. The Danish Register of Causes of Death. Scand J Public Health. 2011;39(7)(suppl):26-29. doi:10.1177/1403494811399958 21775346

[18] Jensen LL, Rohde MC, Banner J, Byard RW. Reclassification of SIDS cases—a need for adjustment of the San Diego classification? Int J Legal Med. 2012;126(2):271-277. doi:10.1007/s00414-011-0624-z 22037935

[19] Winkel BG. Sudden cardiac death in young Danes. Dan Med J. 2012;59(2):B4403. 22293060

[20] Bliddal M, Broe A, Pottegård A, Olsen J, Langhoff-Roos J. The Danish Medical Birth Register. Eur J Epidemiol. 2018;33(1):27-36. doi:10.1007/s10654-018-0356-1 29349587

[21] Pedersen CB. The Danish Civil Registration System. Scand J Public Health. 2011;39(7)(suppl):22-25. doi:10.1177/1403494810387965 21775345

[22] Jensen VM, Rasmussen AW. Danish education registers. Scand J Public Health. 2011;39(7)(suppl):91-94. doi:10.1177/1403494810394715 21775362

[23] Baadsgaard M, Quitzau J. Danish registers on personal income and transfer payments. Scand J Public Health. 2011;39(7)(suppl):103-105. doi:10.1177/1403494811405098 21775365

[24] Oestergaard LB, Christiansen MN, Schmiegelow MD, . Familial clustering of *Staphylococcus aureus* bacteremia in first-degree relatives: a Danish nationwide cohort study. Ann Intern Med. 2016;165(6):390-398. doi:10.7326/M15-2762 27379577

[25] Gundlund A, Christiansen MN, Hansen ML, . Familial clustering and subsequent incidence of atrial fibrillation among first-degree relatives in Denmark. Europace. 2016;18(5):658-664. doi:10.1093/europace/euv274 26559919

[26] Kinney HC, Thach BT. The sudden infant death syndrome. N Engl J Med. 2009;361(8):795-805. doi:10.1056/NEJMra0803836 19692691 PMC3268262

[27] Fleming PJ, Gilbert R, Azaz Y, . Interaction between bedding and sleeping position in the sudden infant death syndrome: a population based case-control study. BMJ. 1990;301(6743):85-89. doi:10.1136/bmj.301.6743.85 2390588 PMC1663432

[28] Moon RY, Horne RSC, Hauck FR. Sudden infant death syndrome. Lancet. 2007;370(9598):1578-1587. doi:10.1016/S0140-6736(07)61662-6 17980736

[29] Blair PS, Sidebotham P, Berry PJ, Evans M, Fleming PJ. Major epidemiological changes in sudden infant death syndrome: a 20-year population-based study in the UK. Lancet. 2006;367(9507):314-319. doi:10.1016/S0140-6736(06)67968-3 16443038

[30] Willinger M, Hoffman HJ, Wu KT, . Factors associated with the transition to nonprone sleep positions of infants in the United States: the National Infant Sleep Position Study. JAMA. 1998;280(4):329-335. doi:10.1001/jama.280.4.329 9686549

[31] Goldstein RD, Trachtenberg FL, Sens MA, Harty BJ, Kinney HC. Overall postneonatal mortality and rates of SIDS. Pediatrics. 2016;137(1). doi:10.1542/peds.2015-2298 26634772

[32] Tursan d’Espaignet E, Bulsara M, Wolfenden L, Byard RW, Stanley FJ. Trends in sudden infant death syndrome in Australia from 1980 to 2002. Forensic Sci Med Pathol. 2008;4(2):83-90. doi:10.1007/s12024-007-9011-y 19291477

[33] Sunekær K, Hansen SH, Banner J. Trends in infant mortality: an evaluation of forensic autopsied infants in Eastern Denmark over 39 years. Int J Legal Med. 2022;136(1):169-178. doi:10.1007/s00414-021-02663-3 34350495

[34] Khoury MJ, Beaty TH, Liang KY. Can familial aggregation of disease be explained by familial aggregation of environmental risk factors? Am J Epidemiol. 1988;127(3):674-683. doi:10.1093/oxfordjournals.aje.a114842 3341366

[35] Blair PS, Fleming PJ, Bensley D, ; Confidential Enquiry into Stillbirths and Deaths Regional Coordinators and Researchers. Smoking and the sudden infant death syndrome: results from 1993-5 case-control study for confidential inquiry into stillbirths and deaths in infancy. BMJ. 1996;313(7051):195-198. doi:10.1136/bmj.313.7051.195 8696194 PMC2351602

[36] Matturri L, Ottaviani G, Lavezzi AM. Maternal smoking and sudden infant death syndrome: epidemiological study related to pathology. Virchows Arch. 2006;449(6):697-706. doi:10.1007/s00428-006-0308-0 17091255

[37] Mitchell EA, Ford RP, Stewart AW, . Smoking and the sudden infant death syndrome. Pediatrics. 1993;91(5):893-896. doi:10.1542/peds.91.5.893 8474808

[38] Opdal SH, Rognum TO. Gene variants predisposing to SIDS: current knowledge. Forensic Sci Med Pathol. 2011;7(1):26-36. doi:10.1007/s12024-010-9182-9 20623341

[39] Winkel BG, Yuan L, Olesen MS, . The role of the sodium current complex in a nonreferred nationwide cohort of sudden infant death syndrome. Heart Rhythm. 2015;12(6):1241-1249. doi:10.1016/j.hrthm.2015.03.013 25757662

[40] Hertz CL, Christiansen SL, Larsen MK, . Genetic investigations of sudden unexpected deaths in infancy using next-generation sequencing of 100 genes associated with cardiac diseases. Eur J Hum Genet. 2016;24(6):817-822. doi:10.1038/ejhg.2015.198 26350513 PMC4867441

[41] Neubauer J, Lecca MR, Russo G, . Post-mortem whole-exome analysis in a large sudden infant death syndrome cohort with a focus on cardiovascular and metabolic genetic diseases. Eur J Hum Genet. 2017;25(4):404-409. doi:10.1038/ejhg.2016.199 28074886 PMC5386419

[42] Ackerman MJ, Siu BL, Sturner WQ, . Postmortem molecular analysis of *SCN5A* defects in sudden infant death syndrome. JAMA. 2001;286(18):2264-2269. doi:10.1001/jama.286.18.2264 11710892

[43] Tester DJ, Wong LCH, Chanana P, . Cardiac genetic predisposition in sudden infant death syndrome. J Am Coll Cardiol. 2018;71(11):1217-1227. doi:10.1016/j.jacc.2018.01.030 29544605

[44] Männikkö R, Wong L, Tester DJ, . Dysfunction of NaV1.4, a skeletal muscle voltage-gated sodium channel, in sudden infant death syndrome: a case-control study. Lancet. 2018;391(10129):1483-1492. doi:10.1016/S0140-6736(18)30021-7 29605429 PMC5899997

[45] Gray B, Tester DJ, Wong LC, . Noncardiac genetic predisposition in sudden infant death syndrome. Genet Med. 2019;21(3):641-649. doi:10.1038/s41436-018-0131-4 30139991

[46] Van Norstrand DW, Ackerman MJ. Sudden infant death syndrome: do ion channels play a role? Heart Rhythm. 2009;6(2):272-278. doi:10.1016/j.hrthm.2008.07.028 18823823 PMC3940066

[47] Tfelt-Hansen J, Winkel BG, Grunnet M, Jespersen T. Cardiac channelopathies and sudden infant death syndrome. Cardiology. 2011;119(1):21-33. doi:10.1159/000329047 21778721

[48] Van Norstrand DW, Ackerman MJ. Genomic risk factors in sudden infant death syndrome. Genome Med. 2010;2(11):86. doi:10.1186/gm207 21122164 PMC3016628

[49] Chahal CAA, Salloum MN, Alahdab F, . Systematic review of the genetics of sudden unexpected death in epilepsy: potential overlap with sudden cardiac death and arrhythmia-related genes. J Am Heart Assoc. 2020;9(1):e012264. doi:10.1161/JAHA.119.012264 31865891 PMC6988156

[50] Ferrante L, Rognum TO, Vege Å, Nygård S, Opdal SH. Altered gene expression and possible immunodeficiency in cases of sudden infant death syndrome. Pediatr Res. 2016;80(1):77-84. doi:10.1038/pr.2016.45 26959483

[51] Ferrante L, Opdal SH. Sudden infant death syndrome and the genetics of inflammation. Front Immunol. 2015;6:63. doi:10.3389/fimmu.2015.00063 25750641 PMC4335605

[52] Tester DJ, Wong LCH, Chanana P, . Exome-wide rare variant analyses in sudden infant death syndrome. J Pediatr. 2018;203:423-428.e11. doi:10.1016/j.jpeds.2018.08.011 30268395 PMC6394853

[53] Tester DJ, Medeiros-Domingo A, Will ML, Haglund CM, Ackerman MJ. Cardiac channel molecular autopsy: insights from 173 consecutive cases of autopsy-negative sudden unexplained death referred for postmortem genetic testing. Mayo Clin Proc. 2012;87(6):524-539. doi:10.1016/j.mayocp.2012.02.017 22677073 PMC3498431

[54] Klaver EC, Versluijs GM, Wilders R. Cardiac ion channel mutations in the sudden infant death syndrome. Int J Cardiol. 2011;152(2):162-170. doi:10.1016/j.ijcard.2010.12.051 21215473

[55] Moon RY; TASK FORCE ON SUDDEN INFANT DEATH SYNDROME. SIDS and other sleep-related infant deaths: evidence base for 2016 updated recommendations for a safe infant sleeping environment. Pediatrics. 2016;138(5):e20162940. doi:10.1542/peds.2016-2940 27940805

[56] Priori SG, Wilde AA, Horie M, . HRS/EHRA/APHRS expert consensus statement on the diagnosis and management of patients with inherited primary arrhythmia syndromes: document endorsed by HRS, EHRA, and APHRS in May 2013 and by ACCF, AHA, PACES, and AEPC in June 2013. Heart Rhythm. 2013;10(12):1932-1963. doi:10.1016/j.hrthm.2013.05.014 24011539

[57] Stiles MK, Wilde AAM, Abrams DJ, . 2020 APHRS/HRS expert consensus statement on the investigation of decedents with sudden unexplained death and patients with sudden cardiac arrest, and of their families. J Arrhythm. 2021;37(3):481-534. doi:10.1002/joa3.12449 34141003 PMC8207384

[58] Saul JP, Schwartz PJ, Ackerman MJ, Triedman JK. Rationale and objectives for ECG screening in infancy. Heart Rhythm. 2014;11(12):2316-2321. doi:10.1016/j.hrthm.2014.09.047 25239430 PMC4254269

[59] Skinner JR, Van Hare GF. Routine ECG screening in infancy and early childhood should not be performed. Heart Rhythm. 2014;11(12):2322-2327. doi:10.1016/j.hrthm.2014.09.046 25239431

[60] Mitchell E, Krous HF, Donald T, Byard RW. An analysis of the usefulness of specific stages in the pathologic investigation of sudden infant death. Am J Forensic Med Pathol. 2000;21(4):395-400. doi:10.1097/00000433-200012000-00020 11111805

[61] Hymel KP; American Academy of Pediatrics; Committee on Child Abuse and Neglect; National Association of Medical Examiners. Distinguishing sudden infant death syndrome from child abuse fatalities. Pediatrics. 2006;118(1):421-427. doi:10.1542/peds.2006-1245 16818592

[62] Tong VT, Jones JR, Dietz PM, D’Angelo D, Bombard JM; Centers for Disease Control and Prevention (CDC). Trends in smoking before, during, and after pregnancy—Pregnancy Risk Assessment Monitoring System (PRAMS), United States, 31 sites, 2000-2005. MMWR Surveill Summ. 2009;58(4):1-29. 19478726

[63] Curtin SC, Matthews TJ. Smoking prevalence and cessation before and during pregnancy: data from the birth certificate, 2014. Natl Vital Stat Rep. 2016;65(1):1-14. 26905977

[64] MacDorman MF, Matthews TJ, Mohangoo AD, Zeitlin J. International comparisons of infant mortality and related factors: United States and Europe, 2010. Natl Vital Stat Rep. 2014;63(5):1-6. 25252091

[65] Alm B, Möllborg P, Erdes L, . SIDS risk factors and factors associated with prone sleeping in Sweden. Arch Dis Child. 2006;91(11):915-919. doi:10.1136/adc.2005.088328 16464961 PMC2082937

[66] Moreno MA. Reducing the risk of sudden infant death syndrome. JAMA Pediatr. 2017;171(2):204. doi:10.1001/jamapediatrics.2016.3097 28166332

