# Supplementary material for: Risk of Sudden Infant Death Syndrome Among Siblings of Children Who Died of Sudden Infant Death Syndrome in Denmark
Source: JAMA Netw Open. 2023 Jan 25;6(1):e2252724. doi: 10.1001/jamanetworkopen.2022.52724 (PMC10187488; doi:10.1001/jamanetworkopen.2022.52724)
Supplement: Supplement 1. — eTable 1. Baseline Characteristics of SIDS Index Cases Compared With the General Pediatric Population During Study Period, 1978-2016 eTable 2. Baseline Characteristics of Siblings of Index Cases Compared With the General Pediatric Population During Study Period, 2002-2016 eTable 3. Baseline Characteristics of SIDS Index Cases Compared With the General Pediatric Population During Study Period 2002-2016 eTable 4. SIRs for SIDS in Siblings of SIDS Index Cases During Study Period 2002-2016 eFigure. Flowchart Illustrating the Selection of the Sibling Cohort, Which Comprised Siblings to Index Cases With SIDS Between 2002 and 2016 [file jamanetwopen-e2252724-s001.pdf]

Supplemental Online Content

Glinge C, Rossetti S, Oestergaard LB, et al. Risk of sudden infant death syndrome among siblings of children who died of sudden infant death syndrome in Denmark. *JAMA Netw Open.* 2023;6(1):e2252724. doi:10.1001/jamanetworkopen.2022.52724

eTable 1. Baseline Characteristics of SIDS Index Cases Compared With the General Pediatric Population During Study Period, 1978-2016

eTable 2. Baseline Characteristics of Siblings of Index Cases Compared With the General Pediatric Population During Study Period, 2002-2016

eTable 3. Baseline Characteristics of SIDS Index Cases Compared With the General Pediatric Population During Study Period 2002-2016

eTable 4. SIRs for SIDS in Siblings of SIDS Index Cases During Study Period 2002-2016

eFigure. Flowchart Illustrating the Selection of the Sibling Cohort, Which Comprised Siblings to Index Cases With SIDS Between 2002 and 2016

This supplemental material has been provided by the authors to give readers additional information about their work.

eTable 1. Baseline Characteristics of SIDS Index Cases Compared With the General Pediatric Population During Study Period, 1978-2016

	Index cases (n=1,465)	General population (n=2,666,834)
Male sex, n (%)	888 (61)	1,395,199 (52)
Age at SIDS, median months (IQR)	3 (2-4)	
Mothers age, median year (IQR)	26 (23-30)	29 (26-33)
**Income (household), n (%)**		
Low	366 (55)	593,580 (33)
Middle	228 (34)	611,575 (34)
High	74 (11)	593,584 (33)
Missing data	797	868,095
**Education (mother), n (%)**		559,802 (26)
Elementary	701 (56)	217,456 (10)
High school	93 (7)	654,019 (31)
Vocational	268 (22)	534,239 (25)
Short/medium higher	153 (12)	161,477 (8)
Long/higher education	30 (2)	539,841 (20)
Missing data	220	1,395,199 (52)

© 2023 Glinge C et al. *JAMA Network Open.*

eTable 2. Baseline Characteristics of Siblings of Index Cases Compared With the General Pediatric Population During Study Period, 2002-2016

**Variables**	**Siblings of index cases (n=122)**	**General population (n=959,140)**	**P-value**
Male sex, n(%)	62 (51)	492,226 (51)	0.91
Mothers age, median year (IQR)	29 (25-34)	31 (27-34)	<0.01
Income (household)*, n(%)			
Low	72 (59)	307,125 (33)	<0.0001
Middle	28 (23)	316,441 (34)	
High	22 (18)	307,130 (33)	
Missing data	0	28,444	
Education (mother), n(%)			
Elementary	65 (59)	151,579 (17)	<0.0001
High school	6 (6)	90,559 (10)	
Vocational	23 (21)	248,971 (28)	
Short/medium/long higher education	17 (15)	387,173 (44)	
Missing data	11	80,858	

*Household income stratified in tertiles low (<495,950 DKK), middle (495,951-720,646 DKK), and high (>720,647).

© 2023 Glinge C et al. *JAMA Network Open.*

eTable 3. Baseline Characteristics of SIDS Index Cases Compared With the General Pediatric Population During Study Period 2002-2016

**Variables**	**Index cases (n=92)**	**General population (n=959,140)**	**P-value**
Male sex, n(%)	55 (60)	492,226 (51)	0.10
Age at SIDS, median months (IQR)	2 (1-4)		
Mothers age, median year (IQR)	27 (23-33)	31 (27-34)	<0.0001
Income (household), n(%)			
Low	60 (65)	307,125 (33)	<0.0001
Middle	24 (26)	316,441 (34)	
High	8 (9)	307,130 (33)	
Missing data	0	28,444	
Education (mother), n(%)			
Elementary	45 (55)	151,579 (17)	<0.0001
High school	5 (6)	90,559 (10)	
Vocational	20 (24)	248,971 (28)	
Short/medium/long higher education	12 (15)	387,173 (44)	
Missing data	10	80,858	

© 2023 Glinge C et al. *JAMA Network Open.*

eTable 4. SIRs for SIDS in Siblings of SIDS Index Cases During Study Period 2002-2016

	**N (no. of events (SIDS))**	**Adjusted SIRs***	**Adjusted SIRs****
General population	959,140 (103)	Reference	Reference
Siblings to SIDS victims	122 (≤3)	3.94 (95% CI 2.55-5.32)	4.17 (95% CI 2.21-6.13)

The reference in the general population. CI=confidence interval. *Adjusted for age, sex and calendar year. ** Adjusted for age, sex, calendar year, mothers age (<30y (median age) vs ≥30y), and mothers education (high school vs. after high school).

© 2023 Glinge C et al. *JAMA Network Open.*

eFigure. Flowchart Illustrating the Selection of the Sibling Cohort, Which Comprised Siblings to Index Cases With SIDS Between 2002 and 2016

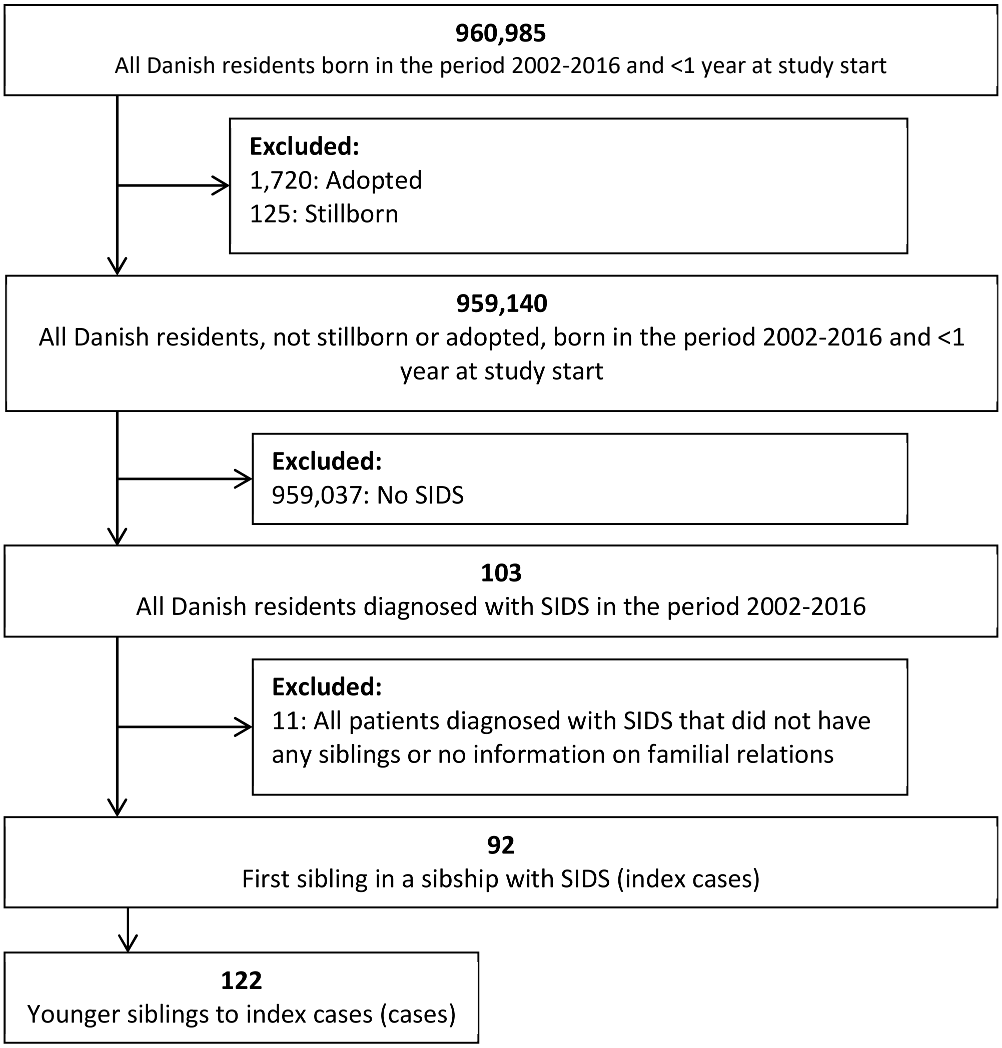

© 2023 Glinge C et al. *JAMA Network Open.*
