# Supplementary material for: Risk of Sudden Infant Death Syndrome Among Siblings of Children Who Died of Sudden Infant Death Syndrome in Denmark
Source: JAMA Netw Open. 2023 Jan 25;6(1):e2252724. doi: 10.1001/jamanetworkopen.2022.52724 (PMC10187488; doi:10.1001/jamanetworkopen.2022.52724)
Supplement: Supplement 2. — Data Sharing Statement [file jamanetwopen-e2252724-s002.pdf]

Data Sharing Statement

Glinge. Risk of Sudden Infant Death Syndrome Among Siblings of Children Who Died of Sudden Infant Death Syndrome in Denmark. *JAMA Netw Open*. Published January 25, 2023. doi:10.1001/jamanetworkopen.2022.52724

Data
Data available: No
